# Endoscopic Endonasal Transsphenoidal Surgery for Recurrent Craniopharyngiomas

**DOI:** 10.3389/fneur.2022.847418

**Published:** 2022-04-11

**Authors:** Zhenguang Feng, Chuzhong Li, Lei Cao, Ning Qiao, Wentao Wu, Jiwei Bai, Peng Zhao, Songbai Gui

**Affiliations:** ^1^Department of Neurosurgery, Tianjin First Center Hospital, Tianjin, China; ^2^Beijing Neurosurgical Institute, Capital Medical University, Beijing, China; ^3^Department of Neurosurgery, Beijing Tiantan Hospital, Capital Medical University, Beijing, China

**Keywords:** craniopharyngiomas, endocrine, endoscopic endonasal transsphenoidal, surgical treatment, recurrent

## Abstract

**Object:**

Although revision surgery for recurrent craniopharyngiomas is more challenging than primary surgery and often accompanies a higher risk of death and complications, endoscopic endonasal transsphenoidal surgery (EETS) is sometimes still an effective and reliable treatment option. In this study, we introduced the surgical outcomes of EETS for recurrent craniopharyngiomas and summarized the surgical experiences.

**Methods:**

Between 2014 and 2018, 28 patients with recurrent craniopharyngiomas underwent 29 EETS in our department. We regarded the patient undergoing two EETS as two independent patients in statistical analysis. Of the 29 patients, 16 had undergone 1 previous surgery, 10 had undergone 2 previous surgeries, and the remaining 3 patients had undergone 3 surgeries. The extent of resection, visual and endocrine outcomes, and complications of all the patients were collected and analyzed.

**Results:**

Gross total resection was accomplished in 16 patients (55.17%), subtotal resection in 11 patients (37.93%), and partial resection in 2 patients (6.9%). Among the 22 patients with preoperative visual acuity and visual field impairment, some degree of vision improvement was observed in 18 patients, 3 patients were without visual change, and perpetual deterioration of vision occurred in one patient. The remaining six patients had normal vision before and after surgery. Postoperative endocrine tests showed that, among five patients with normal preoperative pituitary hormone function, only one patient still had normal pituitary hormone function and the other four patients had one or more hypothalamic-pituitary axes involved. None of the patients with preoperative endocrine dysfunction had endocrine function improved. Diabetes insipidus was observed in six new cases postoperatively. Cerebrospinal fluid (CSF) leakage occurred in 1 patient. One patient had bacterial meningitis, which was cured with antibiotics and a lumbar drain. No serious morbidity and mortality occurred in all patients.

**Conclusions:**

For recurrent craniopharyngiomas, a personalized treatment plan should be developed according to the tumor characteristics and the patient's situation. There is no omnipotent method to be used for all patients. The EETS still is a safe and effective way to treat recurrent craniopharyngiomas in appropriate patients.

## Introduction

Craniopharyngiomas are common benign congenital tumors in the sellar area, which account for about 2–5% of intracranial tumors (1). Adamantinomatous craniopharyngioma (ACP) shows a bimodal age distribution pattern, where the first peak occurs at the age of 5–14 years and a second peak at the age of 40–60 years (2). Conversely, papillary craniopharyngioma (PCP) occurs mainly in adults. There are no significant sex and racial differences (3).

In 1932, craniopharyngioma was described by Cushing as one of the most intractable intracranial tumors (4). To this day, it is still a great challenge for neurosurgeons to perform satisfactory surgical treatment for patients with craniopharyngioma. Although craniopharyngioma is a benign tumor, its deep location and local adherence to the hypothalamus, the optic chiasma, the pituitary gland, three ventricle floor, and other important neurovascular structures make radical resection difficult to achieve (5, 6). Excessive aggressive resection at the expense of pituitary function, hypothalamic or optic injury, may be unacceptable (7).

It is generally believed that gross total resection (GTR) of craniopharyngioma has the potential to be cured (8). The clinical standard for GTR is that complete tumor removal was proved *via* direct visualization by microscope or endoscope in intraoperation and was also confirmed by postoperative MRI. However, the GTR under microscope or endoscope is not equivalent to radical resection of histological and cytological concepts. The residual cells are the origin of recurrences. The more the number of residual tumor cells, the higher is the recurrence rate. Recurrent craniopharyngioma includes the reappearance of tumors or the progression of residual tumors. Compared with primary craniopharyngioma, the surgical treatment of recurrent craniopharyngioma is more difficult (9–11). The previously published reports on endoscopic endonasal transsphenoidal surgery (EETS) for recurrent craniopharyngiomas are limited (6, 10, 12–16). In the present study, we retrospectively analyzed the data of 28 patients who underwent EETS for craniopharyngioma in our department from 2014 to 2018 and summarized the experiences of surgery procedures.

## Methods

A total of 28 patients with recurrent craniopharyngiomas underwent 29 EETS in our department from 2014 to 2018. One of the patients underwent EETS (in 2018) again due to tumor recurrence 2 years after the first EETS (in 2016). We regarded the patient undergoing two EETS as two independent patients in statistical analysis. Patients' medical records and surgical logs were retrospectively analyzed. Follow-ups were conducted in an outpatient setting. This retrospective study was approved by the Ethics Committee of our hospital. All patients were informed of the purpose of this study and signed a written consent form.

### Patients Data

Among the 29 patients, 22 were men, with an average age of ~30 years (ranging from 4 to 58 years); the remaining 7 were women, with an average age of 26.3 years (ranging from 13 to 37 years). Of the 29 patients, 16 had undergone 1 previous surgery, 10 had undergone 2 previous surgeries, and the remaining 3 patients had undergone 3 surgeries. The age at the first surgery ranged from 3 to 46 years (mean 21.7 years). The mean period between patients' most recent surgery and EETS was 58.5 months (range 5–296 months). Twenty-two patients had previously undergone transcranial surgery, four patients had previously undergone the EETS, two patients had previously undergone transsphenoidal microsurgery, and one patient had previously undergone stereotactic aspiration. There were two patients with adjuvant intracystic radiotherapy and four cases with Gamma knife therapy after previous surgery.

### Visual and Endocrine Evaluation

All patients underwent preoperative and postoperative visual field and visual acuity evaluation. Preoperative visual field impairment occurred in 21 patients, and it was normal in 8 patients. All patients underwent endocrine evaluation at our hospital before and after the surgery. Patients with a morning cortisol level and a serum-free thyroxine level below the reference range were deemed to have central adrenal insufficiency and central hypothyroidism. In men, central hypogonadism was diagnosed by low testosterone level with normal or low luteinizing hormone and follicle-stimulating hormone. In women, central hypogonadism was diagnosed according to low or normal gonadotropins accompanied with a low estradiol level, oligomenorrhea, and amenorrhea. Diabetes insipidus (DI) was diagnosed if urine specific gravity was low and polyuria was improved by desmopressin acetate. Of the 29 patients, prior to endoscopic surgery, seventeen were diagnosed as panhypopituitarism, one or two pituitary-target gland axes were involved in seven cases and the remaining five had a normal anterior pituitary function. Fourteen patients were diagnosed to have central DI before endoscopic surgery. After endoscopic surgery, patients underwent blood tests, vision acuity, and vision field examinations at 3 months, 6 months, and 1 year to evaluate endocrine outcomes and visual outcomes.

### Neuroradiological Evaluation

We found that gross total resection (GTR) was obtained in 11 patients, subtotal resection (STR) in 14 patients, and partial resection (PR) in 4 patients in the most recent surgery according to the surgical logs and/or postoperative MRI. The extent of resection (EOR) classifications was as follows: (1) gross total resection (GTR) was defined as a total resection of the tumor with no residual lesion or residual calcification observed in postoperative MRI images, (2) subtotal resection (STR) was defined as ≥90% of the tumor resected in postoperative MRI images, and (3) partial resection (PR) was defined as <90% of the tumor removed in postoperative MRI images.

Preoperative and postoperative MRI scans (the present endoscopic surgery) were performed in the Imaging Department. Imaging characteristics of tumors were identified from preoperative MRI in all cases and included the location and consistency. Tumor volume was calculated using the following equation: V = (D1 x D2 x D3)π/6. This measurement provided only a reasonable estimation rather than an exact measure.

Postoperative MRI was performed in a week to confirm the EOR. MRI images were also obtained 3 months postoperatively and then every 6 months thereafter to observe lesion regrowth and/or recurrence.

The clinical data and the preoperative and postoperative findings for each patient are summarized in Tables 1, 2.

**Table 1:** Summary of preoperative data.

**Case no**.	**Age (years), sex**	**Previous operation**	**Recurrence-free survival (months)^**‡**^**	**Tumor location**	**Tumor consistency**	**Tumor volume (cm^**3**^)**	**Preoperative endocrine**	**Preoperative visual symptoms**
1	M/39	OC	18	Suprasellar	Mixed	11.2	Normal	BV (right)
2	M/21	OC (2 times)	114	Suprasellar	Solid	29.3	Low LH and FSH, TSH; DI	BV (bilateral)
3	M/58	OC	146	Suprasellar	Cystic	9.5	Panhypopituitarism; DI	BV (bilateral)
4	M/44	OC	123	Suprasellar	Solid	35.8	Normal	Normal
5	M/32	OC (2 times) + EET + ICR + γ knife	43	Suprasellar	Mixed	10.6	Panhypopituitarism; DI	BTH
6	M/31	OC	30	Intra- and supra-sellar	Mixed	71.8	Panhypopituitarism; DI	LTH
7	M/33	OC	31	Intra- and supra-sellar	Mixed	12.2	Panhypopituitarism; DI	BV (bilateral)
8	F/37	OC + TMS	9	Intra- and supra-sellar	Solid	175.8	Low TSH, ACTH	BVL
9	M/4	OC	15	Intrasellar	Solid	4.6	Panhypopituitarism; DI	BV (bilateral)
10	M/20	OC (2 times)	20	Intra- and supra-sellar	Mixed	3.6	Low TSH; DI	BV (right)
11	M/17	OC + EET	64	Intra- and supra-sellar	Cystic	18.8	Panhypopituitarism; DI	Normal
12	M/43	TMS + OC	136	Intra- and supra-sellar	Solid	29.6	Panhypopituitarism	BV (right)
13	M/24	OC	110	Intra- and supra-sellar	Cystic	10.4	Normal	BV (bilateral); BTH
14	M/29	OC	62	Suprasellar	Solid	75.8	Low LH and FSH, TSH	Normal
15	M/42	OC (3 times)	68	Intra- and supra-sellar	Solid	15.6	Panhypopituitarism; DI	LVL
16	F/18	OC (2 times)	36	Intrasellar	Solid	4.3	Panhypopituitarism; DI	BV (bilateral)
17*	F/20	OC (2 times) + EET	21	Intra- and supra-sellar	Solid	4.2	Panhypopituitarism	BV (bilateral)
18	M/36	SA + ICR + γ knife	56	Suprasellar	Mixed	18.8	Normal	Normal
19	M/17	OC (2 times)	67	Intra- and supra-sellar; clivus; sphenopalatine sinus	Solid	154.7	Panhypopituitarism	LVL; BV (right)
20	M/30	EET	10	Intra- and supra-sellar	Cystic	6.9	Low ACTH	BV (bilateral)
21	F/29	OC	42	Intra- and supra-sellar	Mixed	42.1	Panhypopituitarism	Normal
22	M/37	OC	296	Suprasellar	Mixed	37	Low LH and FSH; DI	BV (bilateral); BTH
23	M/24	OC (2 times) + γ knife	21	Intra- and supra-sellar	Solid	27.5	Panhypopituitarism	BV (bilateral)
24	M/29	OC (2 times) + γ knife	10	Suprasellar	Solid	34.2	Panhypopituitarism	BV (bilateral); BTH
25	M/43	OC (2 times)	18	Suprasellar	Mixed	25.7	Low TSH, ACTH	BV (bilateral); BTH
26	M/20	OC	14	Intrasellar	Solid	3.8	Panhypopituitarism; DI	BV (bilateral)
27	F/36	OC	44	Suprasellar	Mixed	34.6	Normal; DI	Normal
28	F/13	OC	5	Intra- and supra-sellar	Cystic	20	Panhypopituitarism; DI	Normal
29	M/39	OC	68	Intra- and supra-sellar	Solid	23.6	Panhypopituitarism	BV (left)

OC, open craniotomy; EET, endoscopic endonasal transsphenoidal; ICR, intra-cystic radiotherapy; TMS, transsphenoidal microsurgery; SA, stereotactic aspiration; DI, diabetes insipidus; BV, blurred vision; BTH, bitemporal hemianopia; LTH, left temporal hemianopia; BVL, bilateral vision loss; LVL, left visual loss; The length of Recurrence-free survival was calculated from the patients' most recent surgery to the date of MRI-documented evidence of tumor recurrence or residual tumor progression. ^*^The second endoscopic surgery of Case 16.

**Table 2:** Postoperative data in all the patients.

**Case no**	**EOR**	**Postoperative endocrine outcome**	**Postoperative visual outcome**	**Complications**	**Histology**	**The length of FU(days)**
1	GTR	Panhypopituitarism; DI	Improved	−	ACP	587
2	GTR	Panhypopituitarism; DI	Improved	−	ACP	406
3	GTR	Unchanged	Improved	−	PCP	490
4	GTR	Low TSH, LH	Unchanged	−	ACP	621
5	GTR	Unchanged	Improved	−	ACP	666
6	STR	Unchanged	Improved	−	ACP	733
7	GTR	Unchanged	Improved	−	ACP	1,222
8	PR	Panhypopituitarism; DI	Unchanged	−	MC	1,582
9	STR	Unchanged	Improved	−	ACP	546
10	STR	Panhypopituitarism; DI	Deteriorative	−	ACP	386
11	STR	Unchanged	Unchanged	−	ACP	1,008
12	STR	Unchanged	Improved	−	ACP	1,854
13	STR	Unchanged	Improved	−	PCP	1,159
14	GTR	Panhypopituitarism; DI	Unchanged	−	ACP	1,027
15	STR	Unchanged	Improved	CSF leak	ACP	429
16	GTR	Unchanged	Improved	−	ACP	970
17	GTR	Panhypopituitarism; DI	Improved	−	ACP	371
18	GTR	Low LH and FSH	Deteriorative	BM	ACP	687
19	STR	Unchanged	Improved	−	MC	369
20	GTR	Panhypopituitarism	Improved	−	ACP	1,780
21	GTR	Unchanged	Unchanged	−	ACP	1,575
22	STR	Panhypopituitarism; DI	Improved	−	ACP	810
23	PR	Panhypopituitarism; DI	Unchanged	−	ACP	309
24	GTR	Unchanged	Improved	−	PCP	427
25	GTR	Panhypopituitarism	Improved	−	PCP	433
26	GTR	Unchanged	Unchanged	−	ACP	289
27	STR	Panhypopituitarism; DI	Unchanged	−	ACP	457
28	STR	Unchanged	Unchanged	−	ACP	806
29	GTR	Panhypopituitarism; DI	Improved	−	ACP	649

*ACP, adamantinomatous craniopharyngioma; PCP, papillary craniopharyngioma; MC, malignant craniopharyngioma; CSF leak, cerebrospinal fluid leak; DI, diabetes insipidus; BM, bacterial meningitis*.

### Surgical Procedure

There were differences in the endoscopic endonasal transsphenoidal (EET) procedure for recurrence craniopharyngiomas between the patients with previous craniotomies and the patients with previous EETS OR TSMS. The corridor of the EET approach was a virgin route for patients with previous craniotomies. Therefore, in the description of the surgical process, we divided the patients into two groups according to previous surgical approaches. Group 1 included 23 patients (22 with previous craniotomies, 1 with previous stereotactic aspiration) and Group 2 included 6 patients (4 with previous EET approach and 2 with previous TSM surgery).

### Group 1

**Figure 1:**
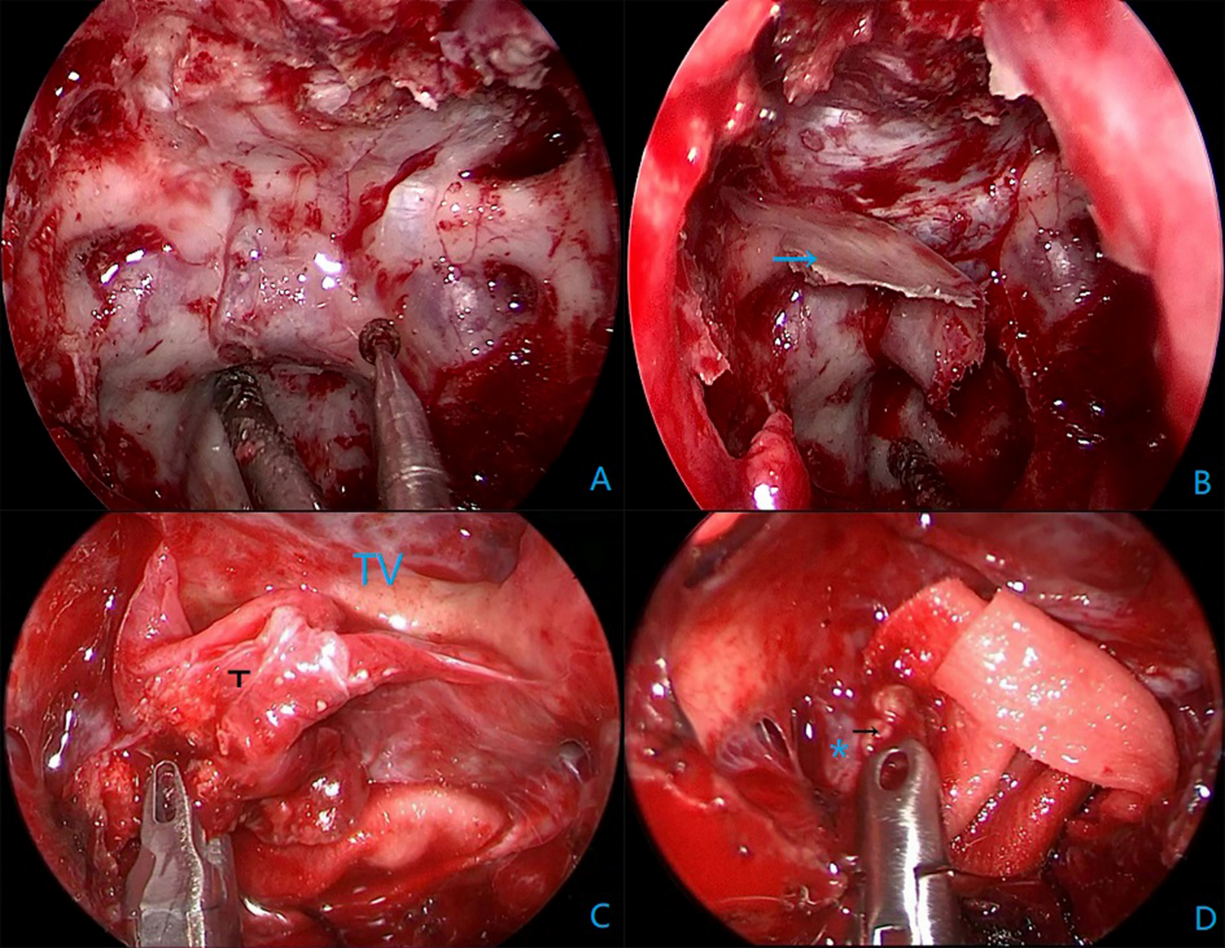
Intraoperative endoscopic views in Case 15. **(A)** Grinding sellar floor anterior to the planum sphenoidal and lateral to the medial optic nerve-internal carotid artery recess. **(B)** A complete bone flap (the blue arrow). **(C)** Tumor (T) and scar adherence to the third ventricular (TV) wall. **(D)** Calcified plaques (the black arrow) adhere tightly to the right posterior communication artery (*).

**Figure 2:**
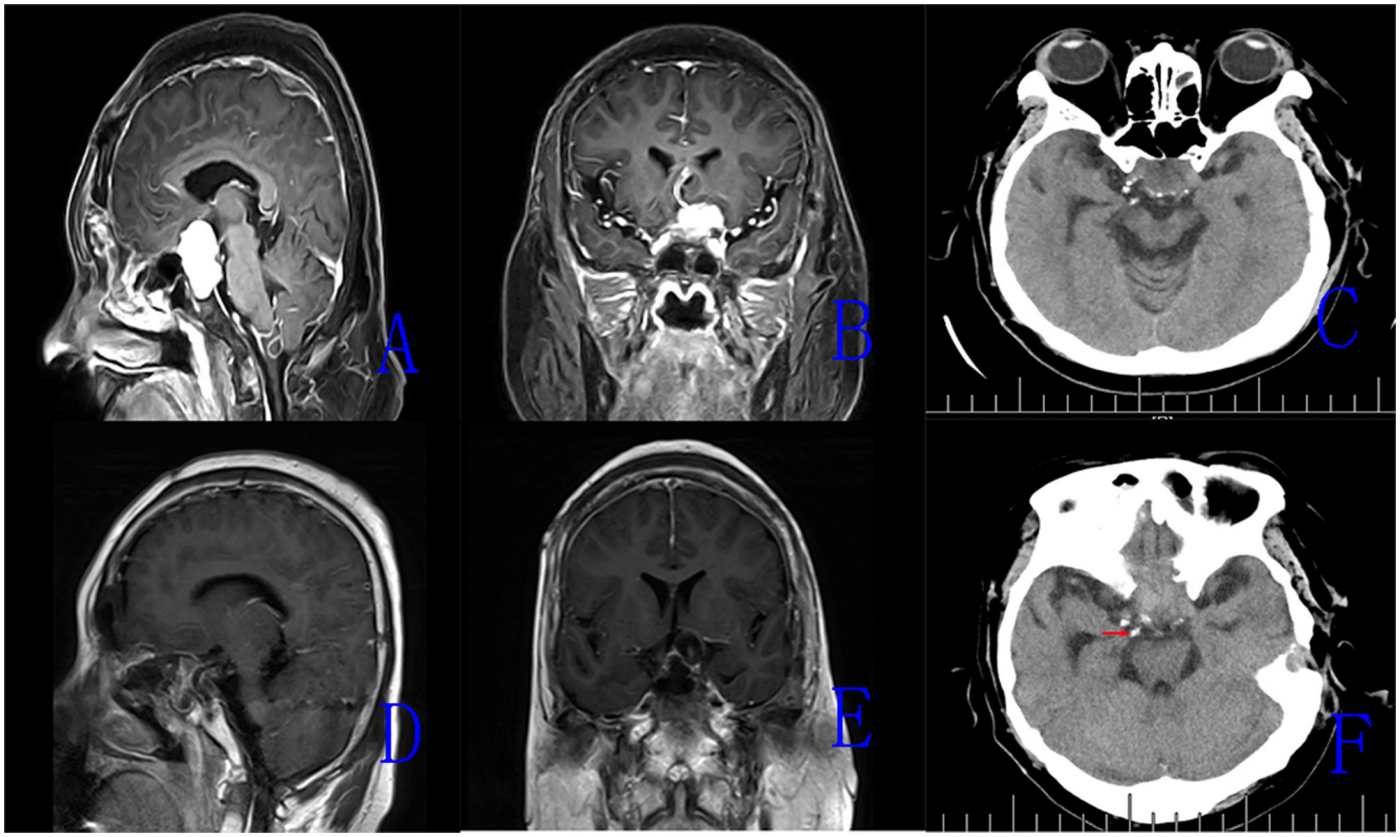
The magnetic resonance imaging (MRI) images of Case 15. **(A,B)** Preoperative enhanced MRI images (sagittal, coronal) showing recurrent intrasellar and suprasellar craniopharyngiomas. **(C)** Preoperative CT scan showing scattered calcified plaques in the tumor. **(D,E)** Postoperative MRI images (sagittal, coronal) demonstrating the tumor removal. **(F)** Postoperative CT scan showing residual calcified plaque of tumor (the red arrow).

All surgical procedures were performed with the patient under general anesthesia. The patient is positioned supine and the head is extended about 10–20°. A rigid 0° endoscope, 18 cm in length and 4 mm in diameter (Karl Storz, Germany) was used in the surgical procedure. In the patients with intrasellar craniopharyngiomas, standard EETS was used. The procedure followed was defined and described in previous publications. In the patients with suprasellar and intra-and suprasellar recurrent craniopharyngiomas, the extended EETS was used. In three extended EETS, the bone of the sella base was removed as a whole anteriorly to the posterior portion of the planum sphenoidale and laterally to the medial optic nerve-internal carotid artery recess (Figure 1). After electrocoagulation of the intercavernous sinus, the dura was opened by a cross-shaped incision. The tumor was removed by gradual debulking. After debulking, the contact surface between the tumor and surrounding tissues can be recognized. On the premise of avoiding further damage to the brain tissue, adhesive tumor tissue was gently removed by the method of sharp separation. Small remnants which were tightly adherent to neurovascular structures were tolerable (Figure 2).

The sellar floor defect was closed in the majority of patients with an absorbable artificial dura, autologous muscle and fat tissue, and a vascularized pedicled septal flap. Finally, the reconstruction was supported by iodoform gauzes. In another 3 patients, the sellar floor reconstruction also included the autologous bone flap.

### Group 2

**Figure 3:**
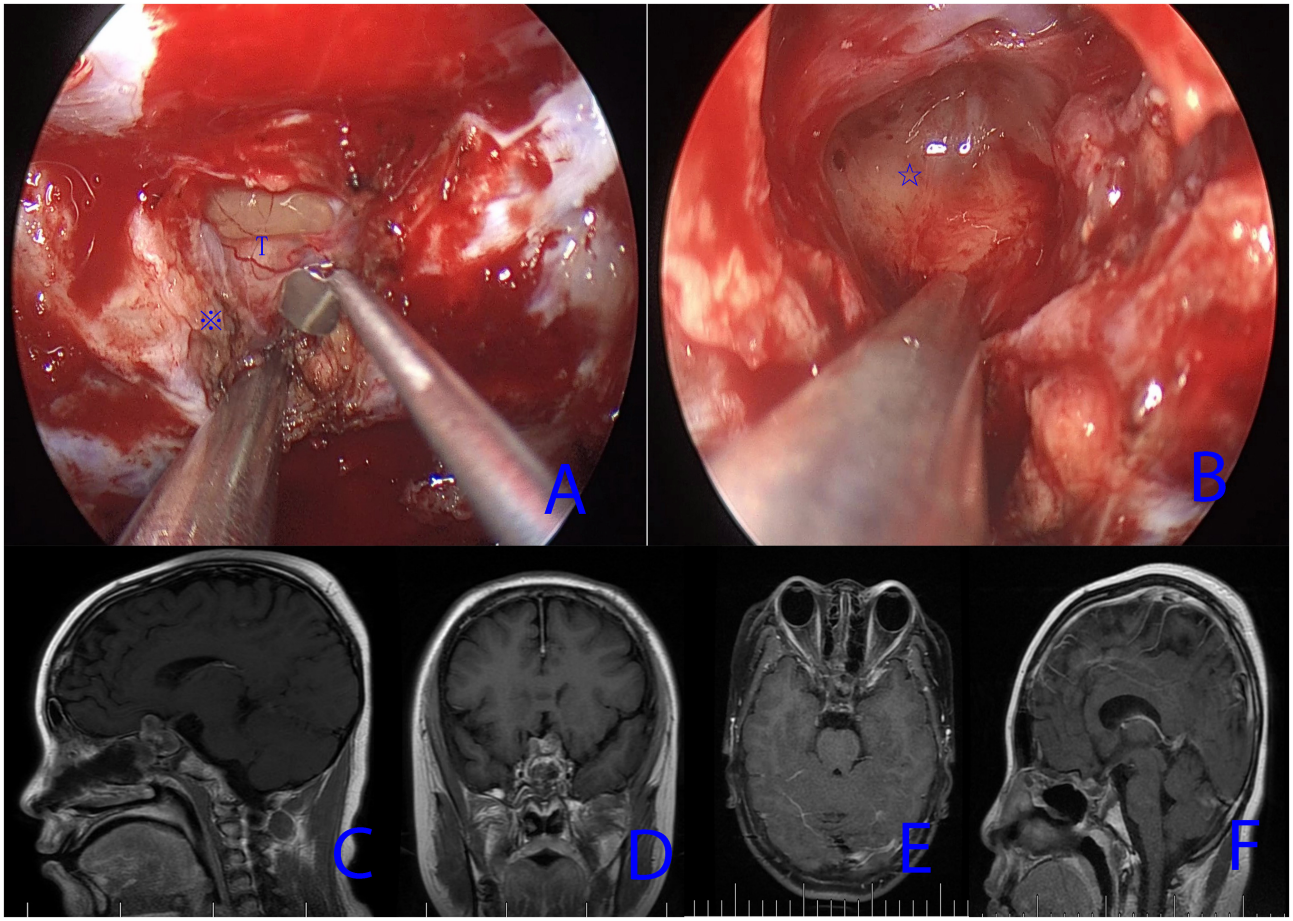
Case 11. **(A,B)** Intraoperative endoscopic views; Asterisk (※) indicates the scar fused with the dura; ⋆ Indicates the thickened arachnoid. T, tumor. **(C,D)** Preoperative MRI images (sagittal, coronal) showing recurrent intrasellar and suprasellar craniopharyngiomas. **(E,F)** Postoperative MRI images (axial, sagittal) demonstrating tumor removal.

Image guidance was used in all patients. An image guidance system could help to maintain the surgeon's orientation and identify anatomic landmarks. In patients with previous EETS, surgical corridors from the nasal cavity to the sphenoid sinus had been established. For the intrasellar recurrent craniopharyngiomas, a wider bilateral sphenoidotomy was performed; for the suprasellar craniopharyngioma and recurrent intrasellar and suprasellar craniopharyngiomas, a transtuberculum–transplanum approach was required. The previous nasal septal flap was opened along the edge of the sellar floor. Then, careful dissection of the hard adhesions and reconstruction materials was required at the sellar floor opening (Figure 3). Sometimes, the position of the internal carotid artery (ICA) was difficult to determine due to the disappearance of the anatomical landmarks. To avoid ICA injury during the full exposure of the tumor, the position of the ICA needed to be determined by intraoperative doppler. In patients with previous transsphenoidal microsurgery, the posterior part of the nasal septum, the middle turbinate, needed to be removed. Further expansion of the sphenoid sinus was performed to obtain a wider exposure of the sellar floor. The tumor resection and skull base reconstruction in the group were the same as described in Group 1.

## Results

A total of 29 patients were enrolled in the present study. The male/female ratio was approximately 3.14/1. The proportion of patients with ACP was significantly higher than that of PCP (23 vs. 4). Although craniopharyngiomas with malignant characteristics were rarely reported, in our series, two malignant craniopharyngiomas were identified (Case 8 and 19). The day of follow-up ranged from 289 to 1,780 (mean 781 days).

According to preoperative neuroradiological findings, solid masses were the most common MRI findings in 14 patients (48.3%), followed by mixed and cystic masses that accounted for 34.5% (10/29) and 17.2% (5/29) of all the patients. A total of 11 patients had purely suprasellar craniopharyngiomas (two extraventricular tumors with remarkable extension into the prepontine cistern and clivus area), 14 patients had intrasellar and suprasellar extension, and 3 patients had intrasellar lesions. In Case 19, the region occupied by the tumor was wide and complex. The enhanced tumor was observed in the sphenoid sinus, intrasellar and suprasellar regions, and clivus area. Moreover, the CT scans of two patients with malignant craniopharyngiomas showed that the bone of sellar turcica was destroyed severely.

The extent of resection for recurrent or progressed residual craniopharyngiomas in the current EETS was confirmed according to intraoperative findings and postoperative 1 week MRI imaging. GTR was accomplished in 16 patients (55.17%), STR in 11 patients (37.93%), and PR in 2 patients (6.9%). In the subgroup of patients who only received previous craniotomies (group 1), GTR was attained in 14 patients, STR in 9 patients, only 1 patient with PR; in the other subgroup (group 2), GTR was attained in 2 patients, STR in 2 patients, and PR in 1 patient. Two patients with malignant craniopharyngiomas were recommended for external radiotherapy 1 month after surgery.

Among the 22 patients with preoperative visual acuity and visual field impairment, some degree of vision improvement was observed in 18 patients, 3 patients were without visual change, and perpetual deterioration of vision occurred in one patient. Case 18, whose preoperative vision was intact, experienced temporary postoperative visual deterioration, and the eye was back to normal within 5 months. The remaining 6 patients had normal vision before and after surgery. Postoperative endocrine tests showed that, among the 5 patients with normal preoperative pituitary hormone function, only 1 patient still had normal pituitary hormone function and the other 4 patients had one or more hypothalamic-pituitary axes involved. None of the patients with preoperative endocrine dysfunction had endocrine function improved. Preoperative DI was unchanged in all 14 patients. Six new cases of postoperative DI were observed.

Cerebrospinal fluid (CSF) leakage occurred in 1 patient one and a half months after endoscopic surgery, and repair operations were performed using the endonasal technique. One patient had bacterial meningitis and was cured with antibiotics and a lumbar drain. No serious morbidity and mortality occurred in any of the patients.

## Discussions

Gross total resection of craniopharyngiomas has been associated with improved long-term outcomes (17–24). Although radical resection should always be attempted, it is not always feasible. Previously published reports showed that GTR rates ranged from 6 to 90% (10, 11, 25–28). The main factors related to the extent of tumor resection were tumor size, hardness, whether with calcification, location, degree of adhesion with surrounding tissues, surgical approach, exposure of the tumor, and experience of the surgeons (29–31). Even when GTR is performed, tumor recurrence occurs potentially. Tumor recurrence has been described in 0–50% of patients who accepted radical surgery, whereas in the STR cases, the recurrence rate can be as high as 30–100% (10, 17, 19, 29). Recurrence rates and mean time to recurrence are dependent on the initial extent of resection and the use of adjuvant radiotherapy (32, 33). These data showed that recurrent craniopharyngioma is a common and unavoidable problem for neurosurgeons.

Several therapeutic modalities have been suggested for recurrent craniopharyngiomas, such as reoperation, external and internal radiotherapy, and chemotherapy; but there is no consensus concerning the optimal treatment for recurrence (4, 31, 34). We believe that the following aspects need to be considered in developing an optimal treatment plan: (1) difficulty and security of reoperation; (2) purely external radiotherapy on recurrent tumor control and its effect on the surrounding important structure; (3) the distribution of internal radiotherapy drugs in the tumor, the effect of cystic wall thickness and tumor calcification on ray penetrating ability, and the risk of radiational damage to adjacent structures; and (4) the side effects of chemotherapy drugs.

In recent years, the EETS has become increasingly popular as a surgical option for the treatment of craniopharyngioma (35–38). Through a straight route, this approach provides a direct visualization to the skull base, which minimizes brain retraction and the manipulation of neurovascular structures. Authors of recent studies have reported patients with craniopharyngiomas who on receiving EETS could obtain satisfying tumor removal rates and long-term surgical outcomes (36–40). Furthermore, the study findings showed that the EETS approach had greater rates of gross total resection, improved visual outcome, and a fewer trend of recurrences than the transcranial approach (35, 37).

Given the advantages and technical progress of EETS in the resection of primary craniopharyngiomas, neurosurgeons have also attempted to use this method to remove recurrent craniopharyngioma. However, compared to the primary craniopharyngioma, the disappearance of a natural cleavage plane formed by a dense gliotic reaction between the tumor and surrounding structures, and the presence of arachnoidal scars from the previous surgery rendered more challenges for the new endoscopic surgery (10). In reports, the authors had underlined the increase of the rates of mortality and morbidity and the decrease of EOR after surgery (12, 15). In 5 years from 2014 to 2018, we treated 29 cases of recurrent craniopharyngiomas by EETS. In our series, GTR was obtained in approximately 55.17% of cases without mortality. With the help of a direct, close-up endoscopic view during surgery, we found that the main causes of STR or PR were tumor tissue with tight adhesion to neurovascular structure (such as optic chiasma, posterior communicating artery, and ICA) and the hypothalamus. Indeed, a small piece of tumor capsule wall adherence to the hypothalamus was residual in 6 patients, and tumor tissue wrapping perforating vessels and posterior communicating arteries was seen in 5 patients. In another 2 patients, calcification plaque was adhering to optic chiasma.

By reviewing the surgical processes of 29 patients, we summarized the steps that need attention and the useful skills in the process of endoscopic surgery for recurrent craniopharyngioma. Before the EETS, a complete understanding of the information of the previous operations, such as surgical approaches, the extent of resection, relations of the residual tumor to surrounding structures, and adjuvant chemoradiotherapy or not was obtained. In addition, ophthalmic and hormone examinations were necessary to fully evaluate the preoperative pituitary endocrine function and visual acuity, and visual field of the patients. A neuroimaging navigation system was a useful tool in recurrent craniopharyngioma surgery, especially in cases who underwent previous transsphenoidal microsurgery or EETS. It could help to maintain the surgeon's orientation and to identify anatomic landmarks to allow for less invasive exposure. When scar and tumor tissues affected the identification of the ICA, an intraoperative doppler could be used to determine the location of the ICA. An intraoperative doppler could reduce the risk of ICA injury. In patients with previous craniotomies, integral bone flap formation was recommended. We believed that a bone flap could provide more support for the sellar floor, and this approach could promote the healing of the nasal septal flap to reduce the risk of CSF leak. In Group 2, when the nasal septal flap was dissected, there may be some bleeding from the scar tissue. Hemostasis was necessary but excessive electrocoagulation should be avoided to prevent septal flap shrinking. Attention should be paid to removing the deepest reconstruction materials to avoid damage to the intradural structures. The tumor adhesion site must be dissected sharply, and blind pulling should be avoided. When encasing tumor tissue or adhering tightly to important neurovascular structures, small residues were acceptable. Furthermore, it was best tried to preserve perforating vessels to the optic nerves and the hypothalamus. In patients with previous EETS, more attention needs to be paid to skull base reconstruction. Removing the scar and expanding the sellar floor opening could cause the area of the mucosal flap to be smaller than the sellar floor defect, and the surgeon was recommended to use meat paste and synthetic protein gum to close the defect. Finally, enough patience was also a necessary quality for the removal of recurrent craniopharyngioma.

Considering the difficulties encountered in new operations for recurrences, it must be prudent to choose endoscopic surgery. The surgeon must be proficient in endoscopic techniques and have extensive experience in the resection of craniopharyngioma.

The short follow-up time was a limitation of the present study. The long-term outcomes and recurrent rate of the patients could not be adequately assessed.

## Conclusions

For recurrent craniopharyngiomas, a personalized treatment plan should be developed according to the tumor characteristics and the patient's situation. There is no omnipotent method to be used for all patients. The EETS still is a safe, effective way to treat recurrent craniopharyngiomas in the appropriate patients.

## Data Availability Statement

The raw data supporting the conclusions of this article will be made available by the authors, without undue reservation.

## Ethics Statement

The studies involving human participants were reviewed and approved by the Ethics Committee of Beijing Tiantan Hospital affiliated to Capital Medical University (Beijing, China). The patients/participants provided their written informed consent to participate in this study.

## Author Contributions

ZF collected and analyzed clinical data and prepared the manuscript. CL designed the trial and revised the manuscript. NQ and WW collected clinical data. SG provided the data revised the manuscript.

## Conflict of Interest

The authors declare that the research was conducted in the absence of any commercial or financial relationships that could be construed as a potential conflict of interest.

## Publisher's Note

All claims expressed in this article are solely those of the authors and do not necessarily represent those of their affiliated organizations, or those of the publisher, the editors and the reviewers. Any product that may be evaluated in this article, or claim that may be made by its manufacturer, is not guaranteed or endorsed by the publisher.
